# A New Tridimensional Insight into Geometric and Kinematic Characteristics of Masticatory Cycles in Participants with Normal Occlusion

**DOI:** 10.1155/2018/2527463

**Published:** 2018-09-03

**Authors:** Ramón Fuentes, Alain Arias, María Florencia Lezcano, Diego Saravia, Gisaku Kuramochi, Pablo Navarro, Fernando José Dias

**Affiliations:** ^1^Department of Integral Adults Dentistry, Dental School, Universidad de La Frontera, Temuco, Chile; ^2^Research Center in Dental Sciences (CICO), Dental School, Universidad de La Frontera, Temuco, Chile; ^3^Universidad Adventista de Chile, Chillán, Chile; ^4^Department of Restorative Dentistry, School of Dentistry, Universidad Finis Terrae, Santiago, Chile; ^5^Universidad Autónoma de Chile, Chile

## Abstract

The aim of this study was to analyze the general, geometric, and kinematic characteristics of the masticatory cycle's movements in a tridimensional way, using a method developed by our study group to provide a new insight into the analysis of mandibular movements due to advancement in the potential of computational analysis. Ten individuals (20.1 ± 2.69 years), molar class I, without mandibular movement problems participated in this study. The movements of the masticatory cycles, frontal and sagittal mandibular border movements, were recorded using 3D electromagnetic articulography and processed with computational scripts developed by our research group. The number of chewing cycles, frequency (cycles/s), chewing cycle areas/mandibular border movements areas ratios, and the mouth opening and closing speeds on the 3D trajectory of the chewing cycle were compared. The cycles were divided and analyzed in thirds. The masticatory cycles showed high variation among the individuals (21.6 ± 9.4 cycles); the frequency (1.46 ± 0.21 cycles/s) revealed a moderate positive correlation (R = 0.52) with the number of cycles. The frontal area ratios between the cycle area and the mandibular border movement presented higher values in the first third (6.65%) of the masticatory cycles, and the ratios of sagittal areas were higher and more variable (first, 7.67%; second, 8.06%; and third, 10.04%) than the frontal view. The opening and closing mouth speeds were greater in the second third of the masticatory cycles (OS, 57.82 mm/s; CS, 58.34 mm/s) without a significant difference between the opening and closing movements when the same thirds were evaluated. Further studies are necessary to improve the understanding of the masticatory cycles regarding the standardization of parameters and their values.

## 1. Introduction

The chewing process is a major step in the digestion of mammals, characterized by a complex motor-sensory activity that consists of rhythmic jaw movements that reduce, grind, and moisten the food, leading to formation of a bolus that can be swallowed. Because of this relationship with digestion and nutritional factors, this functional process is directly related to quality of life [1–6]. The masticatory function integrates the stomatognathic system components, such as the muscles, temporomandibular joint (TMJ), tongue, palate, salivary glands, periodontal, and teeth [7]. Rhythmic jaw movements are referred to as masticatory or chewing cycles; each of these cycles consists of two components: mouth opening and closing [2]. After food has been chopped, it is transported from the anterior region of the mouth to the occlusal surface of the posterior teeth, where crushing and grinding occur for a number of chewing cycles [1, 5]. Although mastication occurs bilaterally, many people present a preferred chewing side [8].

The analysis of the kinematics of human body movements still generates a wide discussion in the literature [9–11]. Among the kinematic characteristics, the movement speed is an important factor that can modify the muscular mechanical characteristics. The improvement in understanding the kinematics of these movements may enhance proposed interventions and consequently their performance [9, 12]. The recording of mandibular movements was introduced into dentistry as a planning tool to analyze the movements' geometry to achieve the best results in treatment [13]. Mandibular movements are complex and occur in three dimensions; however, classical studies have described and analyzed them in two dimensions [14, 15]. In recent decades, technological improvements in position-tracking techniques have enabled the recording of the dynamics of articulations with high temporal resolution [16]. The 3D evaluation of these movements has recently become possible due to the improvement of technology; this kind of analysis allows the evaluation of kinematic (speed) and geometric variables (areas and displacements) of the mandibular movements, including chewing and border movements [14].

Previous studies evaluated the geometric characteristics of the masticatory cycles related to the speed of these movements [15, 17–20] in various clinical situations using different methods of recording and analysis. This fact reveals the importance of a valid quantitative analysis of masticatory movements, which is crucial for dental practice, because it eliminates subjectivity in understanding changes/improvements obtained with clinical interventions [15].

The aim of the present study was to analyze the general, geometrical, and kinesiological characteristics of masticatory cycles in dentate volunteers without functional alterations using a novel electromagnetic 3D-based method developed by the present study group, in order to provide a new insight into the analysis of mandibular movements using a method with promising computational data analysis capability and systematized for translational use at the clinical level.

## 2. Materials and Methods

Ten volunteers, 5 males and 5 females, aged 18-26 years (mean 20.1 ± 2.69 years) were evaluated at the Oral Physiology Laboratory of the Research Center in Dental Sciences (CICO) at the Universidad de La Frontera (Temuco, Chile). All patients agreed to participate through a written informed consent (Ethics Committee Approval no. 038/2016). They showed normal occlusion (normal interarch and dental relationship), molar class I, and complete natural dentition except for the third molar. A screening recommended by the “American Academy of Orofacial Pain” was applied to keep a normal sample without temporomandibular joint disorders. Any neuromuscular-related disease, neurological affectations, orthodontic treatment, or other conditions that could influence the masticatory movements were considered as exclusion criteria.

### 2.1. Mandibular 3D Movement Recordings

The 3D electromagnetic articulography (3D-EMA) is a technique based on the application of the principles of electromagnetic induction that allows the recording of jaw movements, the tongue, and other structures in speaking, swallowing, and chewing research [16, 21–23]. This study used the 3D-EMA AG501 (Carstens Medizinelektronik, Bovenden, Germany) that has high precision and accuracy [24]. To obtain the best performance from the articulograph, the general procedures that were described in the manual and the experimental environment considerations described in our previous work were used [14]. Each subject sat straight and in a comfortable position with their head placed in the center of the magnetic field generated by the articulograph's transmitters. According to the AG501 manual procedures, the head correction function was performed to obtain the normal jaw movements. Five sensors were attached to the subject's head: three were used as a reference system (the left and right mastoid and the glabella) for the other ones, which moved according to this reference system. The other sensors were placed at dynamic points to record the movements of jaw (between the two lower central incisors) and the thyroid cartilage (the cutaneous point). In this manner, the masticatory movements and the end of swallowing were registered.

### 2.2. Task Protocol

All the patients were asked to chew 3.5 g of peanuts, according to previous reports in the literature [19, 25], until swallowing. Mastication was performed 3 times, separated by a rest period of 3 minutes. For each record, mastication started with the patient in maximum intercuspation position (MIP) and with peanuts inside the mouth. The recording finished with the first swallow in order to obtain the number and area of masticatory cycles, the speed of jaw movements (ascending and descending), and the average speed of mastication. Additionally, each patient was instructed to perform the Posselt's envelope of motion [23, 26] in the frontal and sagittal planes before each record of mastication. Thus, the area of each masticatory cycle was compared to the area of each envelope of motion in both the frontal and sagittal planes.

### 2.3. Data Extraction and Analysis

The data files of movements obtained using the 3D-EMA AG501 were processed using computational scripts developed by our research group using MATLAB (MathWorks, Inc., Natick, MA USA). MATLAB is an integrated development environment with its own programming language that is used for signal and imaging processing. This software enabled the visualizing of the movement trajectories recorded and the obtaining of valuable information such as distance, areas, and speeds. The analyzed parameters of the cycles were as follows.

*General Features*

- Number of cycles: The number of cycles performed by the individuals in the 3 replicates was analyzed.
- Frequency: The number of masticatory cycles performed in 1 second (cycles/s) by the individuals in the 3 replicates was analyzed.

The correlation between the number of masticatory cycles and the frequency was also analyzed.

*Geometric Features*. (i) Frontal chewing cycle area/mandibular border movement area ratio: For this parameter, the frontal mandibular border movements were recorded, which were associated with the frontal envelope areas of the masticatory cycles, serving as normalization for the masticatory cycle; thus, it was possible to obtain the percentage ratio of how much of the total area (border) was used in the masticatory movements.

(ii) Sagittal chewing cycle area/mandibular border movement area ratio: The sagittal mandibular border movement envelope areas were associated with the sagittal envelope areas of the masticatory cycles, similar to the frontal view. The normalized percentages of the masticatory cycles that occupied the total area (border) were obtained.

*Kinesiological Features*

- Mouth opening speed: The mean speed (mm/s) of the trajectory of the masticatory cycles during oral opening was analyzed, considering the trajectory of these movements in the 3 planes of the space.
- Mouth closing speed: The mean speed (mm/s) of the trajectory of the masticatory cycles during buccal closure was analyzed, considering the trajectory of these movements in the 3 planes of the space.

The geometric and kinesiological data were separated into thirds from the beginning to the end of chewing (first, second, and third thirds) to make comparisons and to obtain a better understanding of the behavior of these functional movements.

### 2.4. Statistical Analysis

Statistical analysis was performed using SigmaPlot 12.0 software (Systat Software Inc., San Jose, CA, USA). The Shapiro-Wilk test was selected to assess data normality. The quantitative data did not show a normal distribution. We used the Kruskal-Wallis test followed by Dunn's post hoc test, in which case the data are presented as the median, quartile 1 (Q1, 25%), and quartile 3 (Q3, 75%).

## 3. Results

The method of analysis of the masticatory cycles employed in the present study was developed by our research group. This method allows the recording and storage of trajectories of mandibular movements in various motion protocols, including chewing, using a 3D electromagnetic articulograph in an accurate manner.

**Figure 1:**
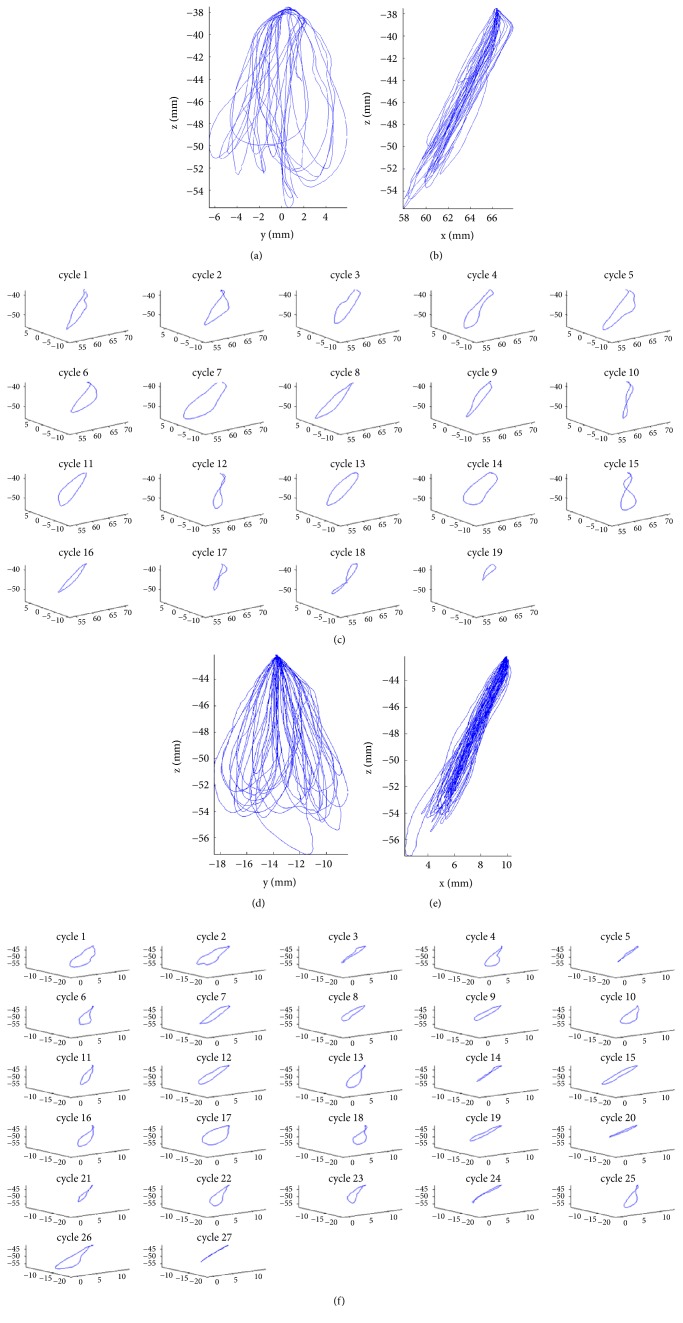
**Masticatory Cycles.** Recorded trajectory of masticatory cycles in female (a, b, and c) and male volunteers (d, e, and f). Front view (a and d), sagittal view (b and e), and individualized cycle trajectories (c and f).

The processing of the data obtained from the recordings using the 3D-EMA AG501 and MATLAB software provided 3D graphics data that enabled the visualization of the envelope images of the trajectories of the masticatory cycles grouped by the frontal view (Figures 1(a) and 1(d)) and the sagittal view (Figures 1(b) and 1(e)) and allowed the individualization of the masticatory cycles (Figures 1(c) and 1(f)). The records of the movements of two patients (patient 1, Figures 1(a), 1(b), and 1(c); patient 2, Figures 1(d), 1(e), and 1(f)) showed differences in the range of movements in both frontal and sagittal views as well as the variation in the number of masticatory cycles whose trajectories were individualized by the script developed for this study; this allowed the 3D evaluation of the trajectory of each masticatory cycle.

The quantitative data of the masticatory cycles were obtained and analyzed (Table 1), and the geometrical and kinesiological features were compared among grouped thirds (first, second, and third) of the chewing cycles.

### 3.1. General Features

**Figure 2:**
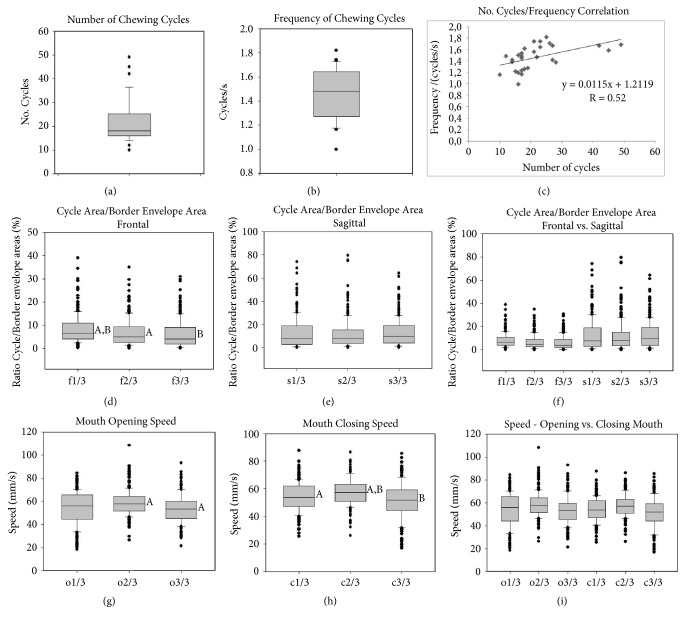
**Graphs of quantitative parameters**. General features: (a) number of chewing cycles, (b) frequency-chewing cycles/second; (c) number of cycles/frequency correlation. Geometrical features: comparison among thirds of chewing cycles, (d) ratio of frontal cycle area/frontal border movements' area, (e) ratio of sagittal cycle area/sagittal border movements' area, (f) frontal x sagittal areas ratio. Kinesiological features: comparison among thirds of chewing cycles, (g) mouth opening speed, (h) mouth closing speed, (i) mouth opening x closing speeds. ^A, B^significant differences (*p* < 0.05).

Among the general characteristics evaluated was the number of masticatory cycles required to chew 3.5 g of peanuts; the mean value of this parameter was 21.6 (± 9.4) cycles and the median was 18 cycles (Q1, 16 cycles; Q3, 25.5 cycles) with values of 10 to 49 cycles (Figure 2(a)). The frequency of the masticatory cycles given by the number of cycles per second showed a mean of 1.46 (± 0.21) cycles/second. The median was 1.48 cycles/s (Q1, 1.27; Q3, 1.65 cycles/s) with variation from 0.99 to 1.82 cycles/second (Figure 2(b)). The correlation between the number of masticatory cycles with their frequency (cycles/s) was moderately positive [27], with a coefficient of 0.52 (Figure 2(c)).

### 3.2. Geometric Features

The mean values of the envelopes of the trajectories of the masticatory cycles and the border areas of the mandibular movements were not compared in this study; however, the data of these parameters are presented in Table 2.

### 3.3. Cycle Area/Border Movement Polygon Area Ratios

The ratio between the frontal areas of the chewing cycles associated with the frontal view mandibular border movement areas in the first third of the masticatory cycles showed a median of 6.65% (Q1, 4.09%; Q3, 11.02%) that was significantly higher (*P* < 0.05) than the ratios of the second (median, 4.99%; Q1; 2.66%; Q3, 9.3% ) and third (median, 4.14%; Q1, 1.85%; Q3, 9.23%) thirds, which were similar (Figure 2(d)).

The ratio between the sagittal areas of the masticatory cycles associated with the sagittal mandibular border movements area did not reveal significant differences (*P* = 0.198) in the comparison of the thirds analyzed; thus, the medians were 7.67% (Q1, 3.07%; Q3, 18.92%) in the first third; 8.06% (Q1, 3.78%; Q3, 15.38%) in the second third; and 10.04% (Q1, 3.99%; Q3, 19.26%) in the third third of the masticatory cycles (Figure 2(e)).

The comparison between the ratios of the areas of the trajectories of the cycles with mandibular border movements through the frontal and sagittal views revealed that a greater percentage of area in the sagittal plane is used on average by the masticatory movements when compared to the percentage of the frontal border area. In addition, a higher variability of these proportions was also revealed by the amplitude of the values in the sagittal view (Figure 2(f)).

### 3.4. Kinesiological Features

The mouth opening speed was significantly higher (P < 0.05) in the second third of the masticatory cycles (median, 57.82; Q1, 51.5; Q3, 64.42 mm/s) in comparison to the third third of the cycles (median, 53.35; Q1, 45.31; Q3, 60.1 mm/s); the mouth opening speed of the first third of the masticatory cycles (median, 56.04; Q1, 44.37; Q3, 65.55 mm/s) did not show a significant difference compared to the other masticatory cycles (Figure 2(g)).

The mouth closing speed was significantly higher (*P* < 0.05) in the second third of the masticatory cycles (median, 57.34; Q1, 50.88; Q3, 63.2 mm/s) when compared to the first (median, 53.81; Q1, 47.24; Q3, 61.88 mm/s) and third (median, 51.94; Q1, 44.28; Q3, 59.37 mm/s) thirds of the cycles (Figure 2(h)).

In the general evaluation between the opening and closing speeds of the masticatory cycles, it was not possible to observe significant differences in the correlates of the thirds in both movements (Figure 2(i)).

## 4. Discussion

The present study evaluated geometric and kinesiological characteristics of masticatory cycles in young individuals without articular and muscle alterations, comparing these characteristics among masticatory cycles grouped in thirds. For this purpose, we used a novel method developed by our research group [14, 23], which employs the 3D electromagnetic articulograph together with the MATLAB software scripts.

The analysis of the images of the trajectories revealed stereotyped movements, mostly a classic elliptical form with the fulcrum in the position of maximum habitual intercuspation. Previous studies using other analysis methods of the masticatory cycle trajectory revealed classic characteristics [15, 18, 19, 26] similar to those observed in the present study. These studies also reported elliptical-shaped cycles, with the fulcrum in the region of maximum intercuspation and sometimes with an “8” shape [15, 18].

The general characteristics of the chewing cycles revealed a wide variation in cycle numbers (21.6 ± 9.4 cycles) due to the differences among the study subjects, even though they were close to 20 years of age with no problems related to mandibular movements. In addition, a moderate positive correlation was observed between the numbers of cycles with their repetition frequency; thus, the higher the number of cycles, the faster their repetition.

Yashiro and colleagues [15, 17] evaluated the characteristics of the masticatory cycles; however, in these cases, the number of cycles that should be performed by the patients was predetermined. We preferred to standardize the evaluation of the masticatory cycles by the same functional activity, the chewing of peanuts, similar to a study performed by Grigoriadis and colleagues [28]; the advantage of this method is the possibility of evaluating chewing efficiency.

The duration of the masticatory cycles previously reported [18, 29] obtained the repetition frequency per second. After a simple conversion, the average values obtained were 1.25 cycles/s [18] and 1.33 cycles/s [29]. The mean and median values in the present study were 1.46 and 1.48 cycles/s, respectively, slightly higher than those previously reported in the literature. This difference may be due to the different ages of the participants, because our study included younger individuals.

Gonçalves and colleagues [19] reported values of the frontal envelope area of the masticatory cycle of 58.5 ± 32.5 mm^2^ and the sagittal envelope of 12.5 ± 2.5 mm^2^. Our results for the frontal and sagittal envelope cycle areas showed lower values in comparison to these means; however, an accordance was noted with a wide variation in the frontal areas. This difference may be related to the different mean ages of the patients, which in our study was ~20.1 years. In the Gonçalves study [19], the patients were over 60 years old and wore total prosthesis, and sets of 20 cycles were recorded and analyzed.

Although the values of the areas of the masticatory cycles are presented in the form of a table (Table 2), our preference was to focus the geometric analysis on the ratio between the areas of the envelopes of the masticatory cycles associated with the envelope of the area of the mandibular movements; we understand that this ratio normalizes the movement of the masticatory cycle. Normalization in this case reduces the inherent differences of the study subjects; for example, if a participant has larger dimensions of the elements involved in the chewing movement, the values of the envelopes areas of the masticatory cycles as well as mandibular border movements are directly affected. Thus, normalizing the masticatory movements using such limits of the movements (border mandibular movements) and evaluating the ratio between the two areas make the analysis more uniform among the different participants of the study. All data collection and processing of these movements were feasible using the method proposed by our study group.

The ratios between the areas of the individual masticatory cycles and the areas of the polygons formed by the frontal and sagittal border movements were grouped in thirds and compared. This comparison was performed to evaluate possible characteristics of this behavior; the first cycles (first third) presented higher values of this ratio compared to the other cycles (second and third thirds). In addition, it was interesting to note that this proportion between the areas using a sagittal view was generally larger and more varied than the frontal view; however, there were no differences between the thirds analyzed.

Several studies have evaluated the speed of the movements of the masticatory cycles [15, 17–20, 28–30]. However, there is still no agreement in reported values, from 36 (± 10.1) mm/s average speed [20] up to 147 mm/s peak speed [30] in patients with healthy teeth. Most of the studies chose a peak speed [17–19, 30, 31] as a base parameter of the speed of masticatory movement, reporting approximate values between 100 and 160 mm/s. Only Amhamed and colleagues [20] used a mean speed varying in a range of 40 to 65 mm/s; the results were still discordant from this study that reported lower speed rates, ~36 to 37 mm/s (SD ~10 mm/s). This disagreement of values may also be due to the different forms of analysis, such as the evaluation of chewing speed in different dental groups (anterior or posterior), in which the different distances from the point of TMJ articulation already generate different displacement values/time; all studies with which we compared our results also evaluated the velocity of the inferior incisor or chin region [15, 17, 19, 20, 29, 30]. Furthermore, our group is working to improve the analysis protocol to include also the evaluation of the angular velocities, which would not be affected by the analyzed region. Also, the type of food or material chewed could also generate differences in the speed analyzed. The choice of peanut for the present study was because it is a more standardized food when compared to a manufactured food, for example, cooked rice and rice cake in [3] and biscuit and bread in [32, 33]; moreover, it is less perishable than other natural foods such as carrots, whose use is also stated in previous reviews [6, 34, 35]. However, most studies evaluating chewing speed prefer to use artificial foods such as gelatins [15, 17, 18, 28, 30] due to the better standardization with different consistencies. Unfortunately, our study group does not rely on these materials to make comparisons more appropriately. However, we justify the use of peanuts also because of the possibility of analysis of general parameters of chewing as a complete physiological action that ends with swallowing; the peanut in this case has the advantage of being a food that can be swallowed.

The kinesiological analysis of the present study revealed median velocities of both buccal opening and closing between 50 and 60 mm/s. The analysis of the velocities of the masticatory cycles grouped in thirds showed the same pattern for the opening and closing velocities of the mouth; thus, the intermediate cycles (second third) are faster both in the opening and especially in the buccal closure. In addition, the thirds (first, second, and third) of the masticatory cycles did not present differences in speed when the opening and closing movements were compared. These studies share the same characteristic of speed evaluation that separated the analyses of opening and closing movements. In addition, our results agree with data from Komagamine and colleagues [18] that reported no significant differences between the opening and closing speeds.

The recording method of mandibular movement using 3D electromagnetic articulography and MATLAB routines revealed graphical, geometric, and kinesiological data similar to other previous studies of 3-dimensional analysis of masticatory movements [15, 17–20, 28–30] and also agreed with the classical observations [26, 36].

However, there is some positive correlation of the parameters analyzed in the present study, but there are still many disagreements in parameter values that need to be better clarified in studies that use standardized parameters. The major advantage of the method of analysis used by our research group is its constant development in search of the best method to customize the analyses carried out based mainly on the objectives that each research requires to seek the most relevant results.

## 5. Conclusion

Using the analysis method developed by the present research group for mandibular movements, it was possible to present a new and updated insight into the analysis of mandibular movements, especially the chewing movements. In addition, it is worth mentioning that the results obtained in this study corroborate the classic studies associated with the theme, reinforcing the previous knowledge and deepening the possibilities of future analyzes. That can be used translationally with studies focused on clinical problems that affect this vital functional action. The analysis of our data allowed us to conclude that the masticatory cycles presented high variation among the young individuals without problems associated with mandibular movement with a moderately positive correlation between the number of cycles and their repetition frequency. The ratio between the envelope area of the cycles and the mandibular border movement area presented higher values in the first third of the masticatory cycles using the frontal view and were larger and more varied using the sagittal view as compared to the frontal view. The speed of opening and closing of the mouth was higher in the third third of the masticatory cycles without revealing differences between the opening and closing movements when the same thirds of cycles were evaluated.

## Figures and Tables

**Table 1:** ** Quantitative data of the masticatory cycles (Part I). **Mean values of the number of masticatory cycles, frequency, and ratios between the area of the cycles and the areas of the border mandibular movements by the frontal and lateral views, and speed of opening and closing of the mouth.

**Patients**	**Gender**	**No. cycles**	**Frequency (cycles/s)**	**Frontal cycle area/border envelope area (**%**)**	**Sagittal cycle area/border envelope area (**%**)**	**Mouth opening speed (mm/s)**	**Mouth closing speed (mm/s)**
1	F	18 ± 1	1.26 ± 0.02	7.13 ± 5.52	10.22 ± 7.22	45.43 ± 9.17	47.32 ± 10.31
2	F	16.3 ± 0.6	1.12 ± 0.1	4.54 ± 4.6	14.95 ± 11.68	46.1 ± 10.05	49.53 ± 8.74
3	F	12.3 ± 2.5	1.29 ± 0.17	14.75 ± 8.81	10.94 ± 9.1	61.29 ± 14.61	44 ± 9.77
4	F	15 ± 1.7	1.42 ± 0.03	3.67 ± 2.4	11.81 ± 9.41	46.68 ± 11.49	44.35 ± 11.44
5	F	25.7 ± 3.2	1.42 ± 0.04	8.56 ± 3.95	11.74 ± 8.91	58.81 ± 10.86	56.25 ± 10.13

6	M	16.5 ± 0.7	1.49 ± 0.01	10.63 ± 10.35	12.3 ± 8.46	62.28 ± 11.87	60.25 ± 7.01
7	M	18.7 ± 2.1	1.57 ± 0.04	12.11 ± 6.38	20.94 ± 20.54	72.36 ± 13.03	67.23 ± 10.53
8	M	24.7 ± 1.5	1.72 ± 0.09	3.62 ± 3.04	11.44 ± 9.55	57.73 ± 11.28	58.46 ± 10.08
9	M	23.7 ± 3.1	1.71 ± 0.04	7.74 ± 6.24	41.78 ± 37	48.91 ± 10.95	56.62 ± 10.67
10	M	45.3 ± 3.5	1.64 ± 0.05	4.95 ± 3.22	4.15 ± 3.36	55.32 ± 8.25	53.86 ± 8.92

	Total	21.6 ± 9.4	1.46 ± 0.21				

**Table 2:** ** Quantitative data of the masticatory cycles (Part II).** Mean values of the frontal and sagittal envelope area of the masticatory cycles and the frontal and sagittal envelope areas of the mandibular border movements.

**Patients**	**Gender**	**Frontal cycle area (mm** ^**2**^ **)**	**Sagittal cycle area (mm** ^**2**^ **)**	**Frontal border envelope area (mm** ^**2**^ **)**	**Sagittal border envelope area (mm** ^**2**^ **)**
1	F	31.96 ± 24.54	8.22 ± 5.81	444.34 ± 35.65	80.47 ± 4.3
2	F	29.13 ± 29.52	10.24 ± 8	642.24 ± 17.12	68.48 ± 25.88
3	F	47.68 ± 28.48	8.97 ± 7.46	323.32 ± 97.83	81.97 ± 40.2
4	F	30.49 ± 19.95	9.74 ± 7.76	830.89 ± 38.14	82.51 ± 25.45
5	F	49.07 ± 22.67	9.61 ± 7.29	573.2 ± 46.38	81.84 ± 11.73

6	M	19.98 ± 19.45	5.06 ± 3.56	187.93 ± 28.03	42.04 ± 13.41
7	M	56.05 ± 29.55	11.72 ± 11.5	463 ± 19.01	55.99 ± 20.5
8	M	24.92 ± 20.91	7.47 ± 6.24	687.84 ± 38.38	65.29 ± 19.04
9	M	20.68 ± 16.67	5.27 ± 4.67	267.09 ± 44.2	12.62 ± 9.17
10	M	24.63 ± 16.04	7.64 ± 6.26	497.66 ± 46.5	185.35 ± 16.5

	Total	32.46 ± 25.02	8.3 ± 7.24		

## Data Availability

The electromagnetic articulography and clinical data used to support the findings of this study have not been made available because we are still employing such data to improve our algorithms.
